# Optimization and comparison of knockdown efficacy between polymerase II expressed shRNA and artificial miRNA targeting luciferase and Apolipoprotein B100

**DOI:** 10.1186/1472-6750-12-42

**Published:** 2012-07-24

**Authors:** Piotr Maczuga, Annemart Koornneef, Florie Borel, Harald Petry, Sander van Deventer, Tita Ritsema, Pavlina Konstantinova

**Affiliations:** 1 Department of Research & Development, uniQure biopharma b.v., Meibergdreef 61, Amsterdam, BA, 1105, The Netherlands; 2 Department of Gastroenterology and Hepatology, Leiden University Medical Center, Leiden, The Netherlands; 3 Department of Gastroenterology and Hepatology, Academic Medical Center, Amsterdam, The Netherlands

## Abstract

**Background:**

Controlling and limiting the expression of short hairpin RNA (shRNA) by using constitutive or tissue-specific polymerase II (pol II) expression can be a promising strategy to avoid RNAi toxicity. However, to date detailed studies on requirements for effective pol II shRNA expression and processing are not available. We investigated the optimal structural configuration of shRNA molecules, namely: hairpin location, stem length and termination signal required for effective pol II expression and compared it with an alternative strategy of avoiding toxicity by using artificial microRNA (miRNA) scaffolds.

**Results:**

Highly effective shRNAs targeting luciferase (shLuc) or Apolipoprotein B100 (shApoB1 and shApoB2) were placed under the control of the pol II CMV promoter and expressed at +5 or +6 nucleotides (nt) with reference to the transcription start site (TSS). Different transcription termination signals (TTS), namely minimal polyadenylation (pA), poly T (T5) and U1 were also used. All pol II- expressed shRNA variants induced mild inhibition of Luciferase reporters carrying specific targets and none of them showed comparable efficacy to their polymerase III-expressed H1-shRNA controls, regardless of hairpin position and termination signal used. Extending hairpin stem length from 20 basepairs (bp) to 21, 25 or 29 bp yielded only slight improvement in the overall efficacy. When shLuc, shApoB1 and shApoB2 were placed in an artificial miRNA scaffold, two out of three were as potent as the H1-shRNA controls. Quantification of small interfering RNA (siRNA) molecules showed that the artificial miRNA constructs expressed less molecules than H1-shRNAs and that CMV-shRNA expressed the lowest amount of siRNA molecules suggesting that RNAi processing in this case is least effective. Furthermore, CMV-miApoB1 and CMV-miApoB2 were as effective as the corresponding H1-shApoB1 and H1-shApoB2 in inhibiting endogenous ApoB mRNA.

**Conclusion:**

Our results demonstrate that artificial miRNA have a better efficacy profile than shRNA expressed either from H1 or CMV promoter and will be used in the future for RNAi therapeutic development.

## Background

RNA interference (RNAi) is an evolutionary conserved mechanism for regulating gene expression. It plays an important role in defense against viruses but also in development and in normal functioning of the cell [1,2]. The natural RNAi mechanism functions by endogenous microRNA (miRNA) molecules, which are synthesized in cells as part of longer primary RNA transcripts (pri-miRNAs). Pri-miRNAs are cleaved by the nuclear Drosha-DGCR8 complex to produce miRNA precursors (pre-miRNAs) of 70 nucleotides (nt), which are then transported by Exportin 5 to the cytoplasm and processed by the RNAse III endonuclease family enzyme Dicer to produce a mature miRNA duplex of ~21,22 basepairs (bp). The guide strand of the miRNA enters a multiprotein RNA-induced silencing complex (RISC) where it is used for sequence-specific recognition of target messenger RNA (mRNA). RISC binding to complementary sequences on the target mRNA results in transcript degradation or translational repression [3].

By introducing molecules that constitute substrates for the endogenous RNAi pathway disease-related mRNA and thus protein translation can be inhibited. RNAi in cells can be induced in different ways. Generally this is achieved by delivery of 20–25 bp-long small interfering RNAs (siRNAs) [4] which serve as substrates for the RISC complex. Alternatively, siRNAs can be generated by expressing short hairpin RNA (shRNA) [5] or artificial microRNA (miRNA) structures [6]. Both enter the endogenous RNAi pathway and are processed into mature siRNAs. The crucial difference between shRNAs and artificial miRNAs is in their secondary structure and processing in the RNAi pathway. shRNAs are normally expressed from polymerase III (pol III) promoters and directly generate a mature product which is exported and processed by Dicer, while miRNAs require an additional step of excision from the longer pre-miRNA sequence by the Drosha-DGCR8 complex. Moreover, miRNAs are expressed from polymerase II (pol II) promoters that allow for the use of tissue-specific or regulated expression systems.

To date, shRNA expression from pol III promoters is the most common way to induce RNAi in cells, which provides potent and stable target inhibition. Several pol III promoters are being used for expression of shRNAs, such as H1 or U6, and transcription initiation and termination sites together with the structural requirements for effective expression of the hairpins are well described [7,8]. However, there are serious disadvantages of pol III expression systems which question their possible application as therapeutic agents. There are cases reporting severe toxicity after administration of high doses of vectors encoding shRNA [9,10]. Toxicity was shown to be associated with oversaturation of the cellular RNAi machinery and changes in endogenous miRNA expression.

This toxicity problem may be circumvented by using weaker pol II promoter to express shRNAs or by embedding siRNA into artificial miRNA scaffolds. The CMV promoter has been the first pol II promoter shown to express active shRNA and initial requirements for this approach have been established: the shRNA has to be juxtaposed to the transcription start site (TSS) (within 6 nt) and followed by the minimal polyadenylation signal (pA) [11]. When these two conditions were not met, the shRNA was found to be inactive. Additionally, to limit toxicity tissue-specific pol II promoters which restrict expression to the target tissue can be applied. This approach has been used to safely express shRNAs targeting JNK1, JNK2, and PI3 under the control of the prostate-specific antigen pol II promoter in androgen-responsive cancer cells [12]. Furthermore, *in vivo* toxicity related to high shRNA expression levels from a pol III promoter was abolished when the shRNA was expressed from the liver-specific pol II hAAT promoter and U1 termination signal was used [13]. Incorporation of a siRNA sequence in an artificial miRNA scaffold has been shown to abolish shRNA-induced neuronal cell death and to avoid disruption of the endogenous RNAi pathway [6,14].

In the current study we focused on the structural requirements to determine the optimal configuration for pol II shRNA expression and compared it to the pol II expression of artificial miRNA. To date, no detailed studies on the hairpin positions, transcription termination signal (TTS), and shRNA stem length requirements for efficient pol II shRNA expression have been performed. Varying positions of the hairpin relative to the TSS +5 or +6, stem length of 20, 21, 25, 29 bp and different TTS (pA, U1 and T5) have been tested for a shRNA targeting luciferase (shLuc) and expressed from the pol II CMV promoter. shLuc at +6 location relative to TSS, with stem length of 20 nt with either pA or U1 termination signal was found to yield optimal results. However, the average luciferase inhibition of 40% was still lower than the control pol III H1-shRNA, which inhibited luciferase up to 90%. Additionally, two shRNAs targeting ApoB (shApoB1 and shApoB2) were found inactive when expressed from CMV using the same configuration. In contrast, when the three shRNAs were incorporated in an artificial miRNA scaffold, two were equally active to their respective H1-shRNA controls while expressing much less siRNA molecules. This indicates that using artificial miRNA scaffolds has a greater therapeutic potential than direct expression of shRNAs from pol II promoters.

## Results

### Design and knockdown efficacy of CMV-shLuc with different transcription start and transcription termination signals

**Figure 1:**
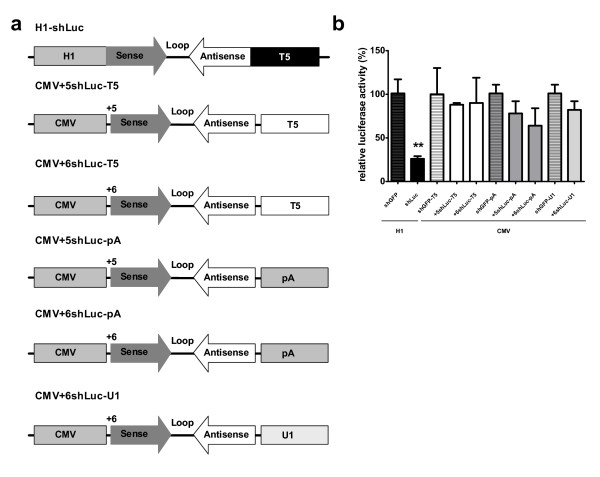
**Structure and knockdown efficacy of short hairpins targeting luciferase (shLuc) with different TSS and different TTS.** (**a**) Schematic representation of shLuc constructs expressed from H1 and CMV promoters. Different TTS (pA, T5 and U1) are presented. Hairpin location (+5 or +6) is shown with reference to the TSS (set as +1). The shLuc consists of 20 nt perfectly complementary hairpin structure and a loop of 9 nt. (**b**) Luciferase knockdown by CMV-shLuc with different TSS and different TTS. Renilla and Firefly luciferase were measured two days post-transfection with 2,5 ng Firefly luciferase reporter, 0,5 ng Renilla luciferase and 100 ng shLuc expressing plasmids. Firefly luciferase expression was normalized to Renilla luciferase expression. H1-shLuc was used as a positive control, H1-shGFP and CMV-shGFP served as negative controls and were set at 100%. Data are represented as mean values ± SD from three independent experiments analyzed with the factor correction method [19]. **p < 0.01 versus negative control One-way ANOVA test with Bonferroni post test.

Previously, we have identified a highly effective siRNA potent in knocking down luciferase when expressed as shRNA from the pol III H1 promoter (shLuc) [15]. To minimize the risk of inducing toxic effects due to siRNA over expression we wanted to express the same shLuc sequence under the control of the pol II CMV promoter. In contrast to pol III, most pol II transcripts undergo posttranscriptional modification at the 5' and 3' ends, which enhances their stability and protects them from degradation [16]. However, both 5’ and 3’ overhangs of the shRNA are important for correct recognition and processing by Dicer [17] therefore these sequences needed to be optimized. A panel of shLuc constructs with perfect complementary 20-bp shRNA stem and 9-nt loop was expressed from the pol II CMV promoter (Figure 1a). Initially, shLuc was placed behind the CMV promoter, followed by a minimal pA signal. The beginning of the passenger strand of the shLuc sequence was placed at positions +5 or +6 relative to the TSS generating CMV + 5shLuc-pA and CMV + 6shLuc-pA, respectively (Figure 1a). It has been reported that T5 TTS can be used instead of pA signal in a CMV-shRNA expression cassette [18]. Therefore, this termination signal was used in the +5shLuc and +6shLuc constructs instead of pA, resulting in CMV + 5shLuc-T5 and CMV + 6shLuc-T5. Finally, the U1 termination signal was cloned behind +6shLuc, resulting in CMV + 6shLuc-U1. To compare their inhibitory effect, HEK293T cells were co-transfected with the different variants of CMV-shLuc together with the Firefly luciferase reporter. shRNA targeting GFP (shGFP) was included as a negative control and Renilla luciferase expression was measured to correct for experimental conditions. The ratio Firefly/Renilla in the negative control was set as 100% and relative inhibition of the luciferase reporter was calculated. All CMV-shLuc constructs appeared less effective in inhibiting luciferase compared to the pol III H1-shLuc control (Figure 1b). The maximum inhibition of luciferase achieved was 22% and 36% for CMV + 5shLuc-pA and CMV + 6shLuc-pA constructs, respectively. CMV + 6shLuc-U1 inhibited luciferase expression with only 18%. CMV + 5shLuc-T5 and CMV + 6shLuc-T5 constructs were not effective at all.

### Design and knockdown efficacy of CMV-shLuc with different hairpin stem length

**Figure 2:**
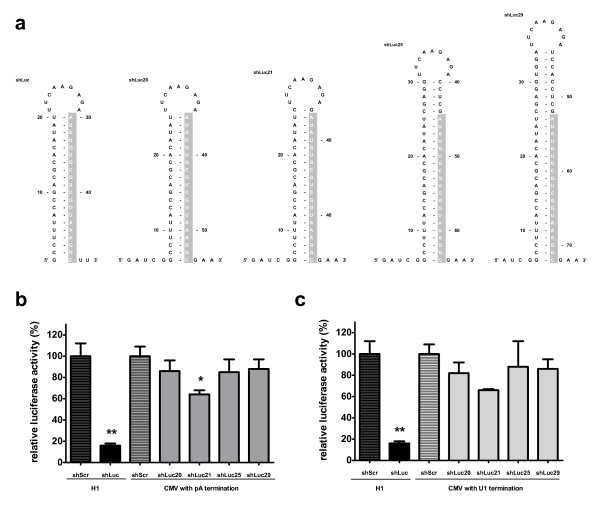
**Structure and knockdown efficacy of CMV-shLuc with different stem length and polyadenylation (pA) or U1 transcription termination signals (U1).** (**a**) Predicted stem-loop structure of CMV-shLuc with different stem lengths (20, 21, 25 and 29 bp). Guide strand is highlighted in grey (**b**) Luciferase knockdown by CMV-shLuc20, CMV-shLuc21, CMV-shLuc25, CMV-shLuc29 with pA transcription termination signal. (**c**) Luciferase knockdown by CMV-shLuc20, CMV-shLuc21, CMV-shLuc25, CMV-shLuc29 with U1 transcription termination signal. Renilla and Firefly luciferase were measured two days post-transfection with 2,5 ng Firefly luciferase reporter, 0,5 ng Renilla luciferase and 100 ng short hairpin expressing plasmid. Firefly luciferase expression was normalized to Renilla luciferase expression. H1-shLuc was used as a positive control. H1-shScr and CMV-shScr served as negative controls and were set at 100%. Data are represented as mean values + SD from three independent experiments analyzed with the factor correction method [19]. *p < 0.05, **p < 0.01 versus negative control (One-way ANOVA test with Bonferroni post test).

The length of the stem in the shRNA has been found to be important for Dicer processing resulting in enhancement of the silencing potential of a given hairpin [20]. Dicer recognizes double-stranded RNA and cleaves 21–22 nt to produce mature siRNA. To check whether extending stem length of shLuc would improve its efficacy as a result of better Dicer processing, constructs with 20, 21, 25 and 29 bp stem were designed by extending the 5’ end of the guide strand, resulting in shLuc20, shLuc21, shLuc25 and shLuc29 variants (Figure 2a). Based on our previous findings shLuc was placed at +6 site and followed by pA or U1 termination signal. Luciferase knockdown was measured to determine the efficacy of the extended stem length constructs (Figure 2b,c). Improved efficacy was observed when extending hairpin stem to 21 bp for both pA (Figure 2b) and U1 TTS (Figure 2c). This improvement was statistically significant for pA TTS (p < 0,05) while change for U1 TTS was not significant (p >0,05). Further lengthening of the shLuc hairpin stem to 25 and 29 bp did not change the inhibitory effect of these constructs (Figure 2b,c).

### Design and knockdown efficacy of CMV-shRNA and artificial miRNA targeting luciferase or Apolipoprotein B100 (ApoB)

**Figure 3:**
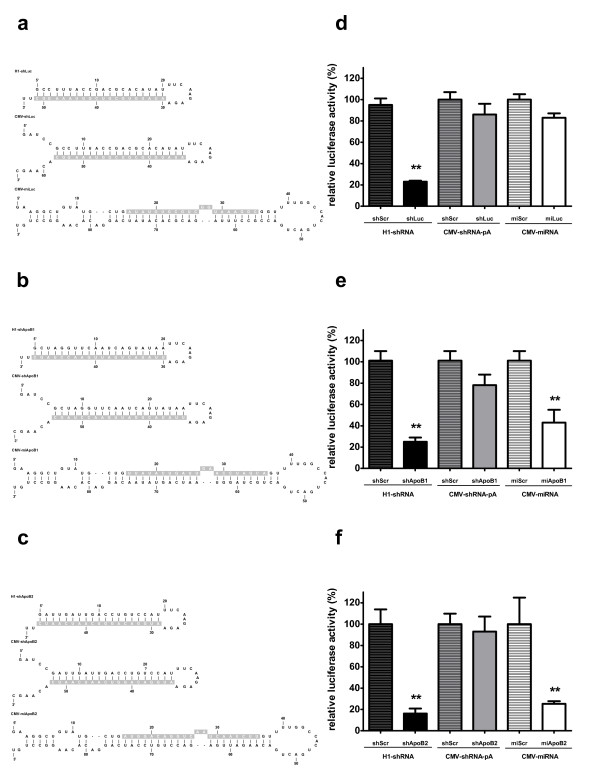
**Structure and knockdown efficacy of shRNA and miRNA hairpin constructs targeting luciferase and Apolipoprotein B100 (ApoB).** (**abc**) Predicted stem-loop structures of shRNA and miRNA targeting luciferase: shLuc or miLuc and ApoB: shApoB or miApoB with guide strand highlighted in grey. shRNA structure is described in Figure 1. miApoB consists of pri-mir-155 precursor sequence, where the mature mir-155 sequence was replaced with the target sequence for luciferase or ApoB. ApoB1 and ApoB2 target different sequences in the ApoB gene. (**d**) Luciferase knockdown by CMV-shLuc and CMV-miLuc. Renilla and Firefly luciferase were measured two days post-transfection with 100 ng shRNA or miRNA expressing plasmid and 2,5 ng Firefly luciferase and 0,5 ng Renilla luciferase. H1-shLuc was used as a positive control. shScr and miScr served as negative controls and were set at 100%. Firefly luciferase expression was normalized to Renilla luciferase expression. Data are represented as mean values ± SD from three independent experiments analyzed with the factor correction method [19] (**e**) Knockdown of Luc-ApoB1 reporter, containing in its 3’ UTR ApoB1 target sequence, by CMV-shApoB1 and CMV-miApoB1. Experimental setup was as described in (**d**) H1-shApoB1 was used as a positive control. (**f**) Knockdown of Luc-ApoB2 reporter, containing in its 3’ UTR ApoB2 target sequence, by CMV-shApoB2 and CMV-miApoB2. Experimental setup was as described in (**d**) with the exception that in this Renilla luciferase contained target sequence for ApoB2 and its expression was normalized to Firefly luciferase expression H1-shApoB2 was used as a positive control. **p < 0.01 versus negative control (One-way ANOVA test with Bonferroni post test).

Previously, H1-shApoB1 and H1-shApoB2 have been used to inhibit *in vitro* and *in vivo* the expression of ApoB that is the structural protein of low density lipoprotein cholesterol (LDL-C) [21] (and data not shown). To evaluate whether the structural requirements for expression of shRNA from the CMV promoter, +6 TSS and pA TTS apply not only to luciferase-targeting constructs, two CMV-shRNAs targeting ApoB were designed and named CMV-shApoB1 and CMV-shApoB2, respectively. As an alternative strategy for direct pol II expression of shRNA, artificial miRNA scaffolds incorporating siRNA targeting luciferase or ApoB were designed by substituting the mature miRNA sequence with luciferase and ApoB target sequences, resulting in CMV-miLuc, CMV-miApoB1 and CMV-miApoB2 (Figure 3a,b,c). The pri-miRNA structure contains additional flanking sequences at the 5' and 3' ends of the hairpin, which enables correct recognition and processing by the RNAi pathway enzymes Drosha and Dicer. The 5' and 3' flanking sequences of pri-mir155 were used, therefore design and optimization of the sequences up- or downstream of the miRNA hairpin are not required for effective knockdown. Knockdown efficacy of CMV-shRNAs and CMV-miRNAs was determined on their specific luciferase reporters, containing ApoB target sequences in case of shApoB and miApoB constructs. H1-shLuc, H1-shApoB1 and H1-shApoB2 were used as positive controls and H1-shScr, CMV-shScr and CMV-miScr, not targeting any sequence in the human genome, were used as negative controls. Luciferase expression in the negative controls was set at 100% and the relative inhibition of luciferase reporters was calculated (Figure 3d,e,f). CMV-shApoB1 and CMV-shApoB2 inhibited luciferase reporters by 22% and 8% respectively (Figure 3e,f), which is similar to the knockdown observed with CMV + 6shLuc20-pA (Figure 3d). By contrast, CMV-miApoB1 and CMV-miApoB2 were found to inhibit luciferase reporters up to 57% and 75% respectively (Figure 3e,f). CMV-miLuc was not more active than CMV + 6shLuc20-pA even though it was designed the same way as CMV-miApoB1 and CMV-miApoB2 (Figure 3d).

### Quantification of siRNA molecules processed from the different shRNA and miRNA scaffolds

**Figure 4:**
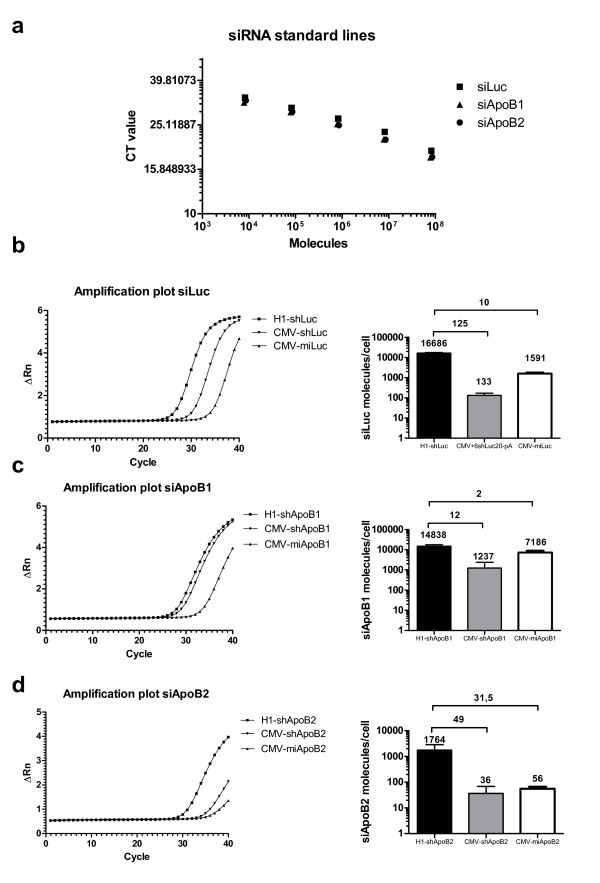
**Quantification of siRNA molecules expressed from H1-shRNA, CMV-shRNA and CMV-miRNA targeting luciferase and Apolipoprotein B100.** (**a**) Synthetic siRNA standard lines. siRNA- specific small RNA TaqMan was performed with dilution series of synthetic siLuc, siApoB1 or siApoB2 molecules. Based on molecular weight of the synthetic siLuc, siApoB1 and siApoB2, the amount of molecules for each point of standard line was calculated and plotted against CT value. (**b**) siLuc amplification plot (left panel) and expression in Hek293T cells (right panel). RNA was isolated two days post-transfection with 1 μg H1-shLuc, CMV-shLuc or CMV-miLuc expressing constructs and siLuc-specific small RNA TaqMan was performed. siRNA copy number was calculated using the synthetic RNA oligo standard line as described in (**a**) (**c**) siApoB1 amplification plot (left panel) and expression in Hek293T cells (right panel) after transfection with 1 μg H1-shApoB1, CMV-shApoB1 or CMV-miApoB1 expressing constructs. Experimental set up as described in (**b**) (**d**) siApoB2 amplification plot (left panel) and expression in Hek293T cells (right panel) after transfection with 1 μg H1-shApoB2, CMV-shApoB2 or CMV-miApoB2 expressing constructs. Experimental set up as described in (**b**). Amplification data are presented from representative experiment from two independent experiments conducted with two technical replicates. siRNA expression data are represented as mean values ± SD from 2 independent experiments conducted with two technical replicates.

Next, we wanted to determine if the inhibitory effect of the shRNA and artificial miRNA constructs correlates with the amount of processed siRNA molecules in the cell. For siLuc, siApoB1 and siApoB2 sequences, small RNA specific TaqMan assay was designed for detecting the guide strand (Additional file 1: Table S1). Ten-fold dilutions (100 pg – 1 fg) of the guide strand of each synthetic siRNA oligo were used to create a standard line (Figure 4a). All siRNA assays showed a clear exponential correlation between the amplification pattern and the amount of siRNA standard. The small RNA TaqMan assay relies on the hybridization of stem-loop RT primers to the mature guide strand and in some cases the method could show insufficient specificity, amplifying the precursor shRNA hairpin or nonspecifically binding to another siRNA molecule. To exclude this possibility, we investigated the ability of the siApoB1 and siApoB2 assays to differentiate between the mature siRNA molecules, their shApoB1 or shApob2 hairpin precursors and expression plasmids by performing additional TaqMan experiments. The primers and probes specifically amplified only the corresponding synthetic siRNA standard and no aspecific amplification was detected for the shRNA hairpin oligo or expression plasmid suggesting that the small RNA TaqMan assays were highly specific for the mature siApoB1 or siApoB2 (Additional file 2: Table S2 and Additional file 3: Table S3) Having confirmed that properly processed siRNA molecules can be specifically detected, we proceeded with detection and quantification of the amount of siRNA molecules per cell for H1-shRNA, CMV-shRNA and CMV-miRNA constructs (Figure 4b,c,d). For all constructs an amplification chart was created and the amount of molecules was calculated. The exponential amplification for H1-shLuc began at 25 cycles, while it began later for both CMV-shLuc and CMV-miLuc (32 and 29 cycles respectively) (Figure 4b left panel). The expression of siLuc molecules was 125-fold lower when expressed as CMV-shLuc and 10-fold lower when expressed as CMV-miLuc compared to H1-shLuc, which resulted in 16686 siRNA molecules per cell (Figure 4b right panel). The exponential amplification for H1-shApoB1 began at 26 cycles, at cycles 32 for CMV-shApoB1 and at 27 cycles for CMV-miApoB1 (Figure 4c left panel). This represents 12-fold less molecules expressed with CMV-shApoB1 and 2-fold less for CMV-miApoB1 compared to H1-shApoB1, which resulted in 14838 siRNA molecules (Figure 4c right panel). siApoB2 molecules were not detected as efficiently as siLuc and siApoB1 thus the exponential amplification started later: for H1-shApoB2, it began at 29 cycles, at 36 cycles for CMV-shApoB2 and at 34 cycles for CMV-miApoB2 (Figure 4d left panel). This represented 49-fold less molecules expressed with CMV-shApoB2 and 32-fold less for CMV-miApoB2 compared to H1-shApoB2, which resulted in 1764 molecules per cell (Figure 4d right panel).

### Endogenous ApoB mRNA knockdown

**Figure 5:**
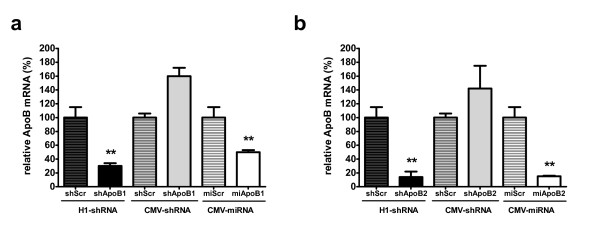
**Comparison of endogenous ApoB mRNA knockdown by shApoB1, shApoB2, miApoB1 and miApoB2in Hepa1-6 cells.** Endogenous ApoB mRNA knockdown by H1-shApoB1, CMV-shApoB1 and CMV-miApoB1 constructs (**b**) Endogenous ApoB mRNA knockdown by H1-shApoB2, CMV-shApoB2 and CMV-miApoB2 constructs qRT-PCR was performed two days post-transfection with 1 μg of shRNA or miRNA expressing constructs and ApoB mRNA levels were calculated relative to actin mRNA. H1-shApoB1 and H1-ApoB1 served as positive control. shScr and miScr served as negative controls and were set at 100%. Data are represented as mean values + SD from three independent experiments analyzed with factor correction method [19]. **p < 0.01 versus negative control (One-way ANOVA test with Bonferroni post test).

In order to assess the inhibitory effect of shApoB1, shApoB2, miApoB1 and miApoB2 constructs on endogenous ApoB expression the mouse hepatoma Hepa1-6 cell line was selected. Cells were transfected with H1-shApoB1, CMV-shApoB1 and CMV-miApoB1 or with their equivalent constructs having ApoB2 as a target. Two days post transfection RNA was isolated and ApoB mRNA expression was determined by RT-qPCR. Both H1-shApoB1 and CMV-miApoB1 efficiently inhibited ApoB mRNA up to 70% and 50% respectively (Figure 5a). An even stronger effect was observed for H1-shApoB2 and CMV-miApoB2 which inhibited ApoB mRNA both up to 85% (Figure 5b). CMV-shApoB1 and CMV-shApoB2 showed no inhibition of endogenous ApoB expression, which confirms the results on luciferase reporters inhibition (Figure 3e,f) and shows the specificity of the designed constructs.

## Discussion

There are few examples of successful shRNA expression using pol II promoters. Although several requirements for pol II shRNA expression have been established, optimization and direct assessment of different hairpin stem lengths, TSS and TTS have not been performed. In the current study we evaluated the relevance of these factors by positioning shRNAs targeting luciferase or ApoB at +5 or +6 relative to the CMV TSS and added the T5, pA or U1 TTS at the 3’ end of the hairpins. Additionally, the same siRNA sequences were incorporated in artificial miRNA scaffolds and direct evaluation between the silencing efficacy and processing of pol II-expressed shRNA and miRNA was performed.

The nucleotide distance between the promoter, the shRNA structure and the TTS determines the length and sequence composition of the 5’ and 3’ overhangs of the expressed hairpin. Both ends act as reference points for recognition and proper processing by Dicer [17]. Initial rules published by Xia *et al.* include shRNA location within 6 nt from the TSS and pA TTS [11]. Later shRNA containing T5 and U1 TTS where also found to be active when substituted for pA [13,18]. These settings were used in our study to express shRNA from the CMV promoter. The shLuc, shApoB1 and shApoB2 sequences have been previously validated as potent inhibitors of their targets when expressed from the pol III H1 promoter [15,21]. Surprisingly, all CMV-shLuc constructs induced only a mild target inhibition when the hairpin was placed at +6 TSS and T5 or pA TTS were used. In contrast to previously published data, the use of U1 TTS did not improve silencing efficacy. By shifting the hairpin from +6 to +5 from TSS, thus minimizing the 5’ overhang, no significant improvement was achieved and the effect was not dependent of TTS used. Additionally, the CMV-shApoB1 and CMV-shApoB2 hairpins were ineffective when the initial settings of +6 TSS and pA TTS were used, indicating that those settings cannot be used as a general rule for pol II shRNA expression.

The length of the shRNA hairpin stem has been shown to play an important role in silencing efficacy as it can lead to differential processing into multiple siRNAs [8,22,23]. In the current study we optimized the pol II shRNA expression by testing stem lengths of 20, 21, 25, and 29 bp. Slight increase in silencing efficacy was observed only when extending hairpin stem from 20 to 21 bp but not when the stem length was further extended to 25 or 29 bp. Mcintyre *et al.* performed a similar study by extending the shRNA stem length from 16 to 41 bp and looked at the core placement of the shRNA [23]. While the processing of hairpins was clearly dependent on the stem length, the activity was primarily dependent on the sequence of processed products. Consistent with their data, our results suggest that there is no strict correlation between the increase in stem length and better silencing activity of the shRNAs. Here, we focused only on pol II expression of shRNA but an alternative approach would be to express long hairpin RNA (lhRNA) where the stem length is extended up to 300 bp [24-26]. Using lhRNA allows generating multiple siRNA from a single transcript, which can be used for viral infections or cancer, where multiple sequences have to be targeted. To date, expression of active lhRNA from pol II promoter has not been successful due to inefficient processing of the hairpins by the RNAi machinery, and detailed studies on TSS, TTS and hairpin location, similar to those for shRNA are lacking.

An alternative to optimizing pol II expression of shRNA is to use pre-miRNA scaffolds and replace the mature miRNA sequence with siRNA targeting a gene of interest. Cellular miRNAs are naturally expressed from pol II promoters [1,27]. Therefore, when shLuc, shApoB1 and shApoB2 were expressed from the pri-mir-155 scaffold the knockdown efficacy was significantly improved. Importantly, artificial miRNA have been found to be less toxic *in vitro* and *in vivo* compared to shRNA as they do not disrupt the endogenous RNAi pathway. McBride *et al.* used this approach to target huntingtin mRNA and to avoid neurotoxicity caused by overexpression of shRNA [14]. Similarly, incorporation of a siRNA against spinocerebellar ataxia 1 protein into an artificial miRNA abolished neuronal cell death observed with a shRNA harboring the same siRNA sequence [6]. In a previous study we have provided evidence for the efficacy of liver-specific expression of artificial miRNA targeting ApoB *in vivo* and demonstrated its advantages over the pol III-expressed shRNA (Maczuga *et al.*, manuscript submitted). In conclusion, we and others have shown that pol II expression of artificial miRNA scaffolds is a more robust and promising approach than pol II shRNA expression, probably due to the specific structural characteristics of the miRNA.

An additional advantage of the use of artificial miRNAs is that several precursors can be expressed as clusters from longer transcripts, allowing simultaneous targeting of multiple genes [28]. This feature of the miRNA is very important when mutation-prone viruses, such as HIV-1 or HCV, are targeted or when several disease-related genes need to be simultaneously silenced. Moreover, miRNAs can be linked to the 3’ untranslated region of a therapeutic gene, which is an additional advantage for therapeutic applications since it allows co-delivery of a codon-optimized gene together with a miRNA targeting the disease-causing version of the same gene. Such combinational therapy has been shown for alpha1-antitrypsin (AAT) deficiency, in which a hairpin RNA targeting mutated AAT transcript was delivered together with codon-optimized AAT gene [29].

Quantification of the siRNA molecules expressed from the H1-shRNA, CMV-shRNA or CMV-miRNA constructs was highly specific and revealed differences in the amount of processed siRNA molecules per cell. As expected, highest siRNA amounts were detected when the shRNAs were expressed from the strong H1 promoter. However, when the same shRNAs were expressed from the CMV promoter, 12- to 125-fold less siRNA molecules were detected indicating that either the shRNA was not efficiently transcribed from the CMV promoter or that there was impaired siRNA processing by the RNAi machinery. Surprisingly, the amount of processed siRNA from the CMV-miRNA constructs did not correlate with efficacy. For example, CMV-miLuc yielded 1591 siLuc molecules per cell and was completely ineffective in target knockdown while CMV-miApoB2 yielded only 56 siApoB2 molecules per cell and was highly efficient. A possible explanation for this discrepancy is that the small RNA TaqMan assay detects only one variant of the guide strand and although identical predicted siRNA sequences were incorporated in the CMV-shRNA and CMV-miRNA scaffolds, they can still be processed differently. Indeed, our newest data based on Next Generation Sequencing (NGS) of small RNAs from cells transfected with H1-shApoB2 and CMV-miApoB2 indicates differential processing and different mature sequences from the two scaffolds (Maczuga *et al.*, manuscript submitted). The siRNAs originating from the H1-shRNA were more heterogeneous in cleavage sites and length compared to the products originating from the CMV-miRNA scaffold supporting the notion that Dicer cleavage is less precise than the combination of Drosha and Dicer. Unfortunately, NGS data from pol II-expressed shRNA, which would allow verifying the processed siRNA variants and their abundance, are still not available.

## Conclusions

In summary, our results demonstrate that it is currently difficult to draw rules for efficient pol II shRNA expression and processing. Optimization of the shRNA stem length, TSS and TTS did not result in better silencing efficacy for any of the CMV-expressed shRNAs. On the other hand, when identical predicted sequences were incorporated in miRNA scaffolds, two out of three sequences resulted in potent inhibition of luciferase reporters and endogenous mRNA. Our data question the application of the previously published rules for pol II shRNA expression as general standards. The rules for design of potent shRNA expressed from pol II promoters are still far from clear and they can be used rather as reference points. Still detailed empirical optimization of hairpin location seems to be required for every target and pol II promoter used. We consider that embedding siRNA in artificial miRNA scaffold and expression from pol II promoter is a better and approach than direct pol II shRNA expression due to the intrinsic characteristics of the miRNA structure rendering it a better substrate for the RNAi machinery.

## Methods

### DNA constructs

H1-shRNAs were made by annealing complementary oligonucleotides and ligating them into the BglII and XhoI sites of the pSuper vector (OligoEngine, Seattle, WA, USA). The vector contains a stretch of five thymidines (T5), which acts as the transcription termination signal. Panel of shRNAs were designed to act as positive controls: one shRNAs was designed that target Firefly luciferase (H1-shLuc), two shRNAs were made that target different regions of human ApoB gene (H1-shApoB1 and H1-shApoB2). Additionally two negative controls shRNA were deigned: one that do not target any gene in human and mouse genome (H1-shScr) and one that target the GFP (H1-shGFP). All the sequences of oligonucleotides used in this study are listed in Additional file 1: Table S1.

CMV-shRNA with different termination signals were designed as followed. First different TTS (T5, pA or U1) were annealed from complementary oligonucleotides and ligated into the AccI and SphI sites of pVD23 which includes the CMV promoter. Next, plasmids were digested with SacI and NheI and complementary oligonucleotides containing shRNA targeting GFP (shGFP) or Luciferase (shLuc) were inserted. shLuc oligonucleotides were designed to allow the hairpin transcription to start at +5 or +6 nucleotides after the TSS, which was considered as +1. The final constructs were CMV + 5shLuc-T5, CMV + 6shLuc-T5, CMV + 5shLuc-pA and CMV + 6shLuc-pA. For U1 termination signal only CMV + 6shLuc-U1 was created. As negative controls CMV-shGFP-T5, CMV-shGFP-pA and CMV-shGFP-U1 were made. GFP oligonucleotides were designed that hairpin started at +6 from the TSS.

CMV-shRNAs with different stem length of the hairpin were designed as followed. CMV-shLuc20, CMV-shLuc21, CMV-shLuc25 or CMV-shLuc29 were made by annealing complementary oligonucleotides that create the stem-loop structure of 20, 21, 25 and 29 bp respectively and ligating them into the BamHI and XhoI sites of the pSilencer 4.1 CMV vector (Life Technologies, Grand Island, NY). Constructs contained either the pA or the U1 termination signal. To create the CMV-shApoB1 and CMV-siApoB2, siRNA sequences from shApoB1 and shApoB2 were cloned by annealing complementary oligonucleotides and ligating them into the BamHI and XhoI sites of the pSilencer 4.1 CMV vector with pA termination signal. As the negative control, pSilencer 4.1 CMV control (Life Technologies, Grand Island, NY) plasmid was used, named CMV-shScr.

To create the artificial miRNA expressing vectors, siRNA sequences from shLuc, shApoB1 and shApoB2 were cloned into the pri-mir-155 backbone of pcDNA6.2-GW/EmGFP-miR (Life Technologies, Grand Island, NY) by annealing complementary oligonucleotides and ligation into BamHI- and XhoI-digested pcDNA6.2 plasmid and named respectively CMV-miLuc, CMV-miApoB1and CMV-miApoB2. As the negative control pcDNA6.2-GW/EmGFP-miR-neg control (Life Technologies, Grand Island, NY) was used, named CMV-miScr. The Mfold program was used to determine the secondary structure of all RNA transcripts [30].

Reporter constructs that were used for luciferase knockdown are Firefly luciferase pGL4 (Promega, Madison, WI) and pRL Renilla (Promega, Madison, WI) under control of the CMV promoter. Luc-ApoB1 reporter, containing in its 3’ UTR a fragment of 239 nucleotides of human ApoB (NM_000384, 9362–9600) sequence was made, by cloning of the ApoB sequence, that was PCR amplified with primers pr565f and pr566r in the XhoI and NheI sites of pGL4 plasmid. For The Luc-ApoB2 containing in the 3’UTR of siCheck2 vector (Promega, Madison, WI) 1851 nucleotides of the last exon from the human ApoB has been described previously.

### Cell culture and transfections

The human embryonic kidney (HEK)293 T and mouse hepatoma Hepa1-6 cell lines were maintained in Dulbecco’s modified Eagle’s medium (DMEM; Life Technologies, Grand Island, NY) containing 10% fetal calf serum, 100 U/ml penicillin and 100 U/ml streptomycin, at 37°C and 5% CO2. For luciferase assays, endogenous ApoB knockdown assays, and siRNA expression analysis, cells were seeded in 96- or 24-well plates at a density of 3*104 or 1.2*105 cells per well, respectively, in DMEM one day prior transfection. Transfections were performed with Lipofectamine 2000 reagent (Life Technologies, Grand Island, NY) according to the manufacturer’s instructions.

### Luciferase assays

Cells were co-transfected with 100 ng shRNA or miRNA expressing plasmid and respectively 2,5 ng Firefly luciferase or Luc-ApoB1 and 0,5 ng Renilla or 50 ng Luc-ApoB2 reporter. Transfected cells were assayed at 48 hr post-transfection in 20 μl 1x passive lysis buffer (Promega, Madison, WI) Firefly and Renilla were measured luciferase activities with the Dual-Luciferase Reporter Assay System (Promega, Madison, WI). For luciferase knockdown calculations, H1-shLuc was used as a positive control. CMV-shScr, CMV-shGFP or CMV-miScr served as negative controls and were set at 100%. For luciferase- and ApoB1- and ApoB1 targeting constructs Firefly luciferase expression was normalized to Renilla luciferase expression. For ApoB2-targeting constructs Renilla luciferase expression was normalized to Firefly luciferase expression since the siCheck2 plasmid was used.

### RNA isolation and quantitative real-time RT-PCR

To determine endogenous ApoB mRNA knockdown by shApoB and miApoB constructs *in vitro*, Hepa1-6 cells were transfected with 1 μg shApoB1, shApoB2, miApoB1 or miApoB2 and total RNA was isolated from cells 48 hr post-transfection using the Nucleospin kit (Clontech, Mountain View, CA). Genomic DNA (gDNA) was removed by DNase treatment using TURBO DNase (Ambion, Austin, TX). First strand cDNA was reverse transcribed using random hexamer primers with the Dynamo kit (Finnzymes, Espoo, Finland). Real time PCR amplification was performed with ApoB- and beta actin-specific primers (Additional file 1: Table S1). PCR reaction conditions were: 95°C for 10 min, followed by 40 cycles of 15 sec at 95°C and 1 min at 60°C. The assays were performed on ABI 7000 or ABI 7500 (Applied Biosystems, Foster City, CA). ApoB expression levels were normalized to beta actin as an internal control, and the relative gene expression 2^-ΔΔCt^ method of Livak and Schmittgen was used for analysis of PCR data [31].

### siRNA detection by small RNA TaqMan assay

RT reactions for siLuc, siApoB1 and siApoB2 expression were performed with the TaqMan MicroRNA Reverse Transcription Kit (Applied Biosystems, Foster City, CA) using 10 ng RNA isolated from cells transfected with shRNA or miRNA expression plasmids and 3 μl custom-made specific RT-stem-loop primers (Applied Biosystems, Foster City, CA) according to the manufacturer’s instructions. The TaqMan assay was done in 20 μl using 1,33 μl cDNA, 1-μl custom-made siRNA-specific primer with FAM-labeled fluorogenic probe (Applied Biosystems) and 10 μl TaqMan 2× Universal PCR Master Mix (Applied Biosystems). For determining the assay specificity, 10 ng of the indicated oligo or expression plasmid was added to the TaqMan reaction. Amplification of the beta actin gene was used as a RNA quality and loading control. siRNA copy number per cell was calculated based on the amplification plot of a dilution series of synthetic siRNA standards (IDT, Coralville, IA). Tenfold dilutions (100 pg – 1 fg) of the guide strand of the synthetic siRNA oligo were used to create a standard line. The copy number of each dilution was calculated according to the formula 1 mol = 6.02 × 10^23^ molecules. The trend line value of the standard line was used to calculate the siRNA copy number per cell, assuming 15 pg RNA per cell [32].

## Competing interests

PM, FB, HP, SvD and PK are employees of uniQure biopharma b.v. AK and TR are former employees of uniQure biopharma b.v. The authors declare that they have no competing interests.

## Authors’ contributions

PM carried out the experiments, designed the study and prepared the manuscript; AK, FB, HP, SvD and TR participated in the study design and drafting of the manuscript; PK designed and interpreted the research and participated in preparing of the manuscript. All authors read and approved the final manuscript.

## Supplementary Material

Additional file 1 **Table S1.** Oligonucleotides used in this study. Click here for file

Additional file 2 **Table S2.** Determination of siApoB1 RT primers and TaqMan probe specificity on siApoB1, shApoB1 hairpin precursor, shApoB1- and miApoB1-expression plasmid and siApoB2. Click here for file

Additional file 3 **Table S3.** Determination of siApoB2 RT primers and TaqMan probe specificity on siApoB2, shApoB2 hairpin precursor, shApoB2- and miApoB2-expression plasmid and siApoB1. Click here for file
