# Supplementary material for: Optimization and comparison of knockdown efficacy between polymerase II expressed shRNA and artificial miRNA targeting luciferase and Apolipoprotein B100
Source: BMC Biotechnol. 2012 Jul 24;12:42. doi: 10.1186/1472-6750-12-42 (PMC3424168; doi:10.1186/1472-6750-12-42)
Supplement: Additional file 1 — Table S1. Oligonucleotides used in this study. [file 1472-6750-12-42-S1.doc]

**Supplementary table 1. Oligonucleotides used in this study**

| **name** | **Sequence (5’-3’)** |
| --- | --- |
| pA forward | agacgctagctaataaaggcatgcttattttcattggatccgtgtgtgttggttttttcctagggcatg |
| pA reverse | ccctaggaaaaaaccaacacacacggatccaatgaaaataagcatgcctttattagctagcgt |
| T5 forward | AGACGCTAGCTATTTTTGGCATGCTTATTTTCATTGGATCCGTGTGTGTTGGTTTTTTCCTAGGGCATG |
| T5 reverse | CCCTAGGAAAAAACCAACACACACGGATCCAATGAAAATAAGCATGCCAAAAATAGCTAGCGT |
| H1-shScr-F | GATCCCCGATCGAATGTGTACTTCGATTCAAGAGATCGAAGTACACATTCGATCTTTTTGCATGCC |
| H1-shScr-R | TCGAGGCATGCAAAAAGATCGAATGTGTACTTCGATCTCTTGGAATCGAAGTACACATTCGATCGGG |
| H1-shGFP-F | GATCCCCAGCTGGAGTACAACTACAACCTTCCTGTCAGTTGTAGTTGTACTCCAGCTTTTTGCATGCC |
| H1-shGFP-R | TCGAGGCATGCAAAAAGCTGGAGTACAACTACAACTGACAGGAAGGTTGTAGTTGTACTCCAGCTGGG |
| CMV+6shGFP-F | CGGTTTAGTCGACCGTCAGATTAGCTGGAGTACAACTACAACTTCAAGAGAGTTGTAGTTGTACTCCAGCTG |
| CMV+6shGFP-R | CTAGCAGCTGGAGTACAACTACAACTCTCTTGAAGTTGTAGTTGTACTCCAGCTAATCTGACGGTCGACTAAACCGAGCT |
| H1-shLucF | GATCCCCGCCTTTACCGACGCACATATTTCAAGAGAATATGTGCGTCGGTAAAGGCTTTTTGCATGCC |
| H1-shLucR | TCGAGGCATGCAAAAAGCCTTTACCGACGCACATATTCTCTTGGAAATATGTGCGTCGGTAAAGGCGGG |
| CMV+6shLucF | CTAGTCAGATTGCCTTTACCGACGCACATATTTCAAGAGAATATGTGCGTCGGTAAAGGCG |
| CMV+6shLucR | CTAGCGCCTTTACCGACGCACATATTCTCTTGAAATATGTGCGTCGGTAAAGGCAATCTGA |
| CMV-shLuc20F | GATCGGCCTTTACTGACGTACATATTTCAAGAGATATGTGCGTCGGTAAAGGCGAA |
| CMV-shLuc20R | AGCTTTCGCCTTTACCGACGCACATATCTCTTGAATATGTACGTCAGTAAAGGCC |
| CMV-shLuc21F | GATCGGCCTTTACTGACGTACATATCTCAAGAGGATATGTGCGTCGGTAAAGGCGAA |
| CMV-shLuc21R | AGCTTTCGCCTTTACCGACGCACATATCCTCTTGAGATATGTACGTCAGTAAAGGCC |
| CMV-shLuc25F | GATCGGCCTTTACTGACGTACATATCGAGGTTCAAGAGCCTCGATATGTGCGTCGGTAAAGGCGAA |
| CMV-shLuc25R | AGCTTTCGCCTTTACCGACGCACATATCGAGGCTTCTTGACCTCGATATGTACGTCAGTAAAGGCC |
| CMV-shLuc29F | GATCGGCCTTTACTGACGTACATATCGAGGTGGATTCAAGAGTCCACCTCGATATGTGCGTCGGTAAAGGCGAA |
| CMV-shLuc29R | AGCTTTCGCCTTTACCGACGCACATATCGAGGTGGACTCTTGATCCACCTCGATATGTACGTCAGTAAAGGCC |
| CMV-shLuc20U1F | GATCGGCCTTTACTGACGTACATATTTCAAGAGATATGTGCGTCGGTAAAGGCGAAGGAGTTTCAAAAGTAGAGCGGCCGCAA |
| CMV-shLuc20U1R | TGCGGCCGCTCTACTTTTGAAACTCCTTCGCCTTTACCGACGCACATATCTCTTGAATATGTACGTCAGTAAAGGCC |
| CMV-shLuc21U1F | GATCGGCCTTTACTGACGTACATATCTTCAAGAGGATATGTGCGTCGGTAAAGGCGAAGGAGTTTCAAAAGTAGAGCGGCCGCAA |
| CMV-shLuc21U1R | AGCTTTGCGGCCGCTCTACTTTTGAAACTCCTTCGCCTTTACCGACGCACATATCCTCTTGAGATATGTACGTCAGTAAAGGCC |
| CMV-shLuc25U1F | GATCGGCCTTTACTGACGTACATATCGAGGTTCAAGAGCCTCGATATGTGCGTCGGTAAAGGCGAAGGAGTTTCAAAAGTAGAGCGGCCGCAA |
| CMV-shLuc25U1R | AGCTTTGCGGCCGCTCTACTTTTGAAACTCCTTCGCCTTTACCGACGCACATATCGAGGCTCTTGACCTCGATATGTACGTCAGTAAAGGCC |
| CMV-shLuc29U1F | GATCGGCCTTTACTGACGTACATATCGAGGTGGATTCAAGAGTCCACCTCGATATGTGCGTCGGTAAAGGCGAAGGAGTTTCAAAAGTAGAGCGGCCGCAA |
| CMV-shLuc29U1R | AGCTTTGCGGCCGCTCTACTTTTGAAACTCCTTCGCCTTTACCGACGCACATATCGAGGTGGA CTCTTGA TCCACCTCGATATGTACGTCAGTAAAGGCC |
| CMV-miLucF1 | GATCCTGGAGGCTTGCTGAAGGCTGTATGCTGATATGTGCGTCGGTAAAGGCGGTTTTGGCCACTGACTGACCGC |
| CMV-miLucF2 | CTTTAGACGCACATATCAGGACACAAGGCCTGTTACTAGCACTCACATGGAACAAATGGCCCAGATCTGGCCGCAG |
| CMV-miLucR1 | TCGACTGCGGCCAGATCTGGGCCATTTGTTCCATGTGAGTGCTAGTAACAGGCCTTGTGTCCTGATATGTGCGTCTAAAGGCGGTCAGTCAGTGGCCAAAACC |
| CMV-miLucR2 | GCCTTTACCGACGCACATATCAGCATACAGCCTTCAGCAAGCCTCCAG |
| H1-shApoB1F | GATCCCCGCTAGGTTTAATTAGTATGATTCAAGAGATTATACTGATTGAACCTAGCTTTTTGCATGCC |
| H1-shApoB1R | TGCAAAAAGCTAGGTTCAATCAGTATAATCTCTTGAATCATACTAATTAAACCTAGCGGG |
| CMV-shApoB1F | GATCGGCTAGGTTCAATCAGTATAAGTCAAGAGCTTATACTGATTGAACCTAGCACA |
| CMV-shApoB1R | AGCTTGTGCTAGGTTCAATCAGTATAAGCTCTTGACTTATACTGATTGAACCTAGCC |
| CMV-miApoB1F1 | GATCCTGGAGGCTTGCTGAAGGCTGTATGCTGTTATACTGATTGAACCTAGCAGTTTTGGCCACTGACTGACTGC |
| CMV-shApoB1F2 | TAGGTAATCAGTATAACAGGACACAAGGCCTGTTACTAGCACTCACATGGAACAAATGGCCCAGATCTGGCCGCAG |
| CMV-miApoB1R1 | TCGACTGCGGCCAGATCTGGGCCATTTGTTCCATGTGAGTGCTAGTAACAGGCCTTGTGTCCTGTTATACTGATTACCTAGCAGTCAGTCAGTGGCCAAAACT |
| CMV-shApoB1R2 | GCTAGGTTCAATCAGTATAACAGCATACAGCCTTCAGCAAGCCTCCAG |
| H1-shApoB2f | GATCCCCGATTGATTGACCTGTCCATTTCAAGAGAATGGACAGGTCAATCAATCTTTTTC |
| H1-shApoB2r | TCGAGAAAAAGATTGATTGACCTGTCCATTCTCTTGAAATGGACAGGTCAATCAATCGGG |
| CMV-shApoB2F | GATCTGATTGATTGACCTGTCCATTCTCAAGAGGAATGGACAGGTCAATCAATCTTA |
| CMV-shApoB2R | AGCTTAAGATTGATTGACCTGTCCATTCCTCTTGAGAATGGACAGGTCAATCAATCA |
| CMV-miApoB2F1 | GATCCTGGAGGCTTGCTGAAGGCTGTATGCTGATGGACAGGTCAATCAATCTTGTTTTGGCCACTGACTGACAAG |
| CMV-miApoB2F2 | ATTGAGACCTGTCCATCAGGACACAAGGCCTGTTACTAGCACTCACATGGAACAAATGGCCCAGATCTGGCCGCAG |
| CMV-miApoB2R1 | TCGACTGCGGCCAGATCTGGGCCATTTGTTCCATGTGAGTGCTAGTAACAGGCCTTGTGTCCTGATGGACAGGTCTCAATCTTGTCAGTCAGTGGCCAAAACA |
| CMV-miApoB2R2 | AGATTGATTGACCTGTCCATCAGCATACAGCCTTCAGCAAGCCTCCAG |
| Pr565f | CAGCTCGAGTCCCAGTGCCCAGCAAGC |
| Pr566r | CGTGCTAGCCTGTTTTTTCCCATAGAG |
| ApoBf | TGGTTACTGCGCTGAGAGG |
| ApoBr | CCACACTGAACCAAGGCTT |
| m-act-f | ACGGCCAGGTCATCACTATTG |
| m-act-r | CAAGAAGGAAGGCTGGAAAAGA |
| shApoB1 oligo | GATCCCCGCTAGGTTTAATTAGTATGATTCCAAGAGATTATACTGATTGAACCTAGCTTTTTC |
| miApoB1 oligo | TTATACTGATTGAACCTAGCAGTTTTGGCCACTGACTGACTGCTAGGTAATCAGTATAACAGG |
| shApoB2 oligo | GATCCCCGATTGATTGACCTGTCCATTTCAAGAGAATGGACAGGTCAATCAATCTTTTTC |
| miApoB2 oligo | ATGGACAGGTCAATCAATCTTGTTTTGGCCACTGACTGACAAGATTGAGACCTGTCCATCAGG |
