# Supplementary material for: Optimization and comparison of knockdown efficacy between polymerase II expressed shRNA and artificial miRNA targeting luciferase and Apolipoprotein B100
Source: BMC Biotechnol. 2012 Jul 24;12:42. doi: 10.1186/1472-6750-12-42 (PMC3424168; doi:10.1186/1472-6750-12-42)
Supplement: Additional file 2 — Table S2. Determination of siApoB1 RT primers and TaqMan probe specificity on siApoB1, shApoB1 hairpin precursor, shApoB1- and miApoB1-expression plasmid and siApoB2. [file 1472-6750-12-42-S2.doc]

Supplementary table 2. Determination of siApoB1 RT primers and Taqman probe specificity on siApoB1, shApoB1 hairpin precursor, shApoB1- and miApoB1-expression plasmid and siApoB2

| Sample | Amount | RT primer | Taqman probe | CT value | Molecules per cell |
| --- | --- | --- | --- | --- | --- |
| siApoB1 synthetic standard | 10 pg | ApoB1 | ApoB1 | 15,1 | 1,23E+05 |
| siApoB1 synthetic standard | 1 pg | ApoB1 | ApoB1 | 17,2 | 1,23E+04 |
| siApoB1 synthetic standard | 0,1 pg | ApoB1 | ApoB1 | 21,5 | 1,23E+03 |
| siApoB1 synthetic standard | 0,01 pg | ApoB1 | ApoB1 | 24,9 | 1,23E+02 |
| siApoB1 synthetic standard | 0,001pg | ApoB1 | ApoB1 | 29,0 | 1,23E+01 |
| siApoB1 synthetic standard | 10 pg | ApoB2 | ApoB1 | ND | ND |
| siApoB1 synthetic standard | 1 pg | ApoB2 | ApoB1 | ND | ND |
| siApoB1 synthetic standard | 0,1 pg | ApoB2 | ApoB1 | ND | ND |
| siApoB1 synthetic standard | 0,01 pg | ApoB2 | ApoB1 | ND | ND |
| siApoB1 synthetic standard | 0,001pg | ApoB2 | ApoB1 | ND | ND |
| siApoB2 synthetic standard | 10 pg | ApoB1 | ApoB1 | ND | ND |
| siApoB2 synthetic standard | 1 pg | ApoB1 | ApoB1 | ND | ND |
| siApoB2 synthetic standard | 0,1 pg | ApoB1 | ApoB1 | ND | ND |
| siApoB2 synthetic standard | 0,01 pg | ApoB1 | ApoB1 | ND | ND |
| siApoB2 synthetic standard | 0,001pg | ApoB1 | ApoB1 | ND | ND |
| H1-shApoB1 plasmid | 10 ng | - | ApoB1 | ND | ND |
| CMV-shApoB1 plasmid | 10 ng | - | ApoB1 | ND | ND |
| CMV-miApoB1 plasmid | 10 ng | - | ApoB1 | ND | ND |
| shApoB1 oligo | 10 ng | - | ApoB1 | ND | ND |
| miApoB1 oligo | 10 ng | - | ApoB1 | ND | ND |

siApoB1- specific assay was tested against synthetic RNA standards, shApoB1-expression plasmids and shApoB1 hairpin oligonucleotides using different configuration of RT primer and Taqman probe. Average CT values lower than 40 cycles are presented. The amount of molecules per cell was calculated, assuming that 15 pg RNA was isolated per cell [33]. Data are presented from one representative experiment from two independent experiments conducted with two technical replicates. ND, not detected
