# Supplementary material for: Optimization and comparison of knockdown efficacy between polymerase II expressed shRNA and artificial miRNA targeting luciferase and Apolipoprotein B100
Source: BMC Biotechnol. 2012 Jul 24;12:42. doi: 10.1186/1472-6750-12-42 (PMC3424168; doi:10.1186/1472-6750-12-42)
Supplement: Additional file 3 — Table S3. Determination of siApoB2 RT primers and TaqMan probe specificity on siApoB2, shApoB2 hairpin precursor, shApoB2- and miApoB2-expression plasmid and siApoB1. [file 1472-6750-12-42-S3.doc]

Supplementary table 3. Determination of siApoB2 RT primers and Taqman probe specificity on siApoB2, shApoB2 hairpin precursor, shApoB2- and miApoB2-expression plasmid and siApoB1

| Sample | Amount | RT primer | Taqman probe | CT value | Molecules per cell |
| --- | --- | --- | --- | --- | --- |
| siApoB2 synthetic standard | 10 pg | ApoB2 | ApoB2 | 12,7 | 1,28E+05 |
| siApoB2 synthetic standard | 1 pg | ApoB2 | ApoB2 | 16,2 | 1,28E+04 |
| siApoB2 synthetic standard | 0,1 pg | ApoB2 | ApoB2 | 19,4 | 1,28E+03 |
| siApoB2 synthetic standard | 0,01 pg | ApoB2 | ApoB2 | 23,2 | 1,28E+02 |
| siApoB2 synthetic standard | 0,001pg | ApoB2 | ApoB2 | 23,7 | 1,28E+01 |
| siApoB2 synthetic standard | 10 pg | ApoB1 | ApoB2 | 28,7 | 7,13E+00 |
| siApoB2 synthetic standard | 1 pg | ApoB1 | ApoB2 | 32,0 | 5,26E-01 |
| siApoB2 synthetic standard | 0,1 pg | ApoB1 | ApoB2 | 36,4 | 1,72E-02 |
| siApoB2 synthetic standard | 0,01 pg | ApoB1 | ApoB2 | ND | ND |
| siApoB2 synthetic standard | 0,001pg | ApoB1 | ApoB2 | ND | ND |
| siApoB2 synthetic standard | 10 pg | ApoB2 | ApoB1 | ND | ND |
| siApoB2 synthetic standard | 1 pg | ApoB2 | ApoB1 | ND | ND |
| siApoB2 synthetic standard | 0,1 pg | ApoB2 | ApoB1 | ND | ND |
| siApoB2 synthetic standard | 0,01 pg | ApoB2 | ApoB1 | ND | ND |
| siApoB2 synthetic standard | 0,001pg | ApoB2 | ApoB1 | ND | ND |
| H1-shApoB2 plasmid | 10 ng | - | ApoB2 | ND | ND |
| CMV-shApoB2 plasmid | 10 ng | - | ApoB2 | ND | ND |
| CMV-miApoB2 plasmid | 10 ng | - | ApoB2 | ND | ND |
| shApoB2 oligo | 10 ng | - | ApoB2 | ND | ND |
| miApoB2 oligo | 10 ng | - | ApoB2 | ND | ND |

siApoB2- specific assay was tested against synthetic RNA standards, shApoB2-expression plasmids and shApoB1 hairpin oligonucleotides using different configuration of RT primer and Taqman probe. Average CT values lower than 40 cycles are presented. The amount of molecules per cell was calculated, assuming that 15 pg RNA was isolated per cell [33]. Data are presented from one representative experiment from two independent experiments conducted with two technical replicates. ND, not detected
